# The crisis of surgical vocations: burnout, generational shifts, and the future of general surgery

**DOI:** 10.3389/fsurg.2026.1812678

**Published:** 2026-04-13

**Authors:** Andrea Cavallaro, Antonio Zanghì, Alessandro Cappellani, Paolo Di Mattia, Francesco Leonforte, Kenya Tiralongo

**Affiliations:** 1 General Surgery III, Department of General Surgery and Medical-Surgical Specialties, University of Catania, AOU Policlinico “G. Rodolico - San Marco”, Catania, Italy; 2 General Surgery III Unit, ChiSMaCoTA Research Center, Department of General Surgery and Medical-Surgical Specialties, AOU Policlinico “G. Rodolico - San Marco”, Catania, Italy; 3 General Surgery III Unit, Department of General Surgery and Medical-Surgical Specialties, AOU Policlinico “G. Rodolico - San Marco”, Catania, Italy; 4 General Surgery, Umberto I Hospital, Department of Medicine and Surgery, Kore University, Enna, Italy; 5 Department of Integrated Hygiene, Organizational, and Service Activities (Structural Department), Health Management, University Hospital Policlinico “G. Rodolico-San Marco”, Catania, Italy

**Keywords:** factors influencing career choice in surgery, gender disparity, professional crisis, surgeon burnout, surgical vocation crisis, surgical workforce trends, work-life balance

## Introduction

The term ‘professional crisis’ or ‘vocational crisis’ in surgery refers to a situation in which general surgery residents increasingly choose to specialize in a particular surgical or medical subspecialty, rather than pursuing a career as a general surgeon.

The ancient Confucian teaching “Choose a job you love, and you will never have to work a day in your life” echoes with particular intensity in the world of surgery. For generations, surgeons have embraced this ideal, merging vocation, discipline, and technical mastery into a unified ethical identity.

Historically, young physicians entering surgery perceived themselves as part of a lineage, inheriting the values of craftsmanship, courage, altruism, and humility from their mentors. The operating room was not merely a workplace but a symbolic environment where technical mastery intertwined with moral duty.

Yet today, the landscape has dramatically changed. The traditional harmony between calling, competence, and identity is increasingly disrupted. What older generations of surgeons viewed as the natural progression from apprentice to master now appears uncertain, fragmented, and in many cases unsustainable. The modern trainee encounters not only the inherent demands of surgical practice but also a healthcare environment characterized by structural constraints, administrative overload, and shifting cultural expectations. The very notion of “vocation,” once synonymous with sacrifice and devotion, is increasingly contested by younger physicians who prioritize balance, well-being, and long-term quality of life. This combination has contributed to what is now often described as a crisis of surgical vocation, a phenomenon with significant implications for recruitment, retention, and the future of the entire discipline (1).

This article provides an opinion-based perspective supported by a focused narrative review of the literature. Relevant studies were identified through searches of PubMed/MEDLINE and Google Scholar, prioritizing systematic reviews, meta-analyses, and large surveys addressing surgeon burnout, surgical workforce trends, and factors influencing career choice in surgery over the past decade. Additional relevant sources were identified through cross-referencing of key publications.

## Burnout among healthcare professionals

Over the last two decades, a marked decline in the attractiveness of general surgery among medical graduates has been observed, reflecting two closely related and partially overlapping phenomena: reduced specialty appeal and attrition during surgical training or professional practice. The former is largely driven by evolving generational expectations regarding lifestyle balance, career sustainability, and professional fulfillment, whereas the latter is more closely associated with occupational stress, unfavorable working conditions, and burnout-related factors.

Burnout is well-characterized in the literature and is typically defined by the triad of emotional exhaustion, depersonalization, and diminished personal effectiveness.

Emotional exhaustion reflects physical and emotional depletion; depersonalization involves negative, detached reactions to work; reduced efficacy denotes feelings of incompetence or low self-esteem. Individuals experiencing burnout are depleted, impairing work performance. With rising rates, exacerbated by the COVID-19 pandemic, burnout in medicine is now considered a public health crisis (1). A recent review by Park and McElveen linked burnout to the deterioration of physicians’ physiological health behaviors, emphasizing the importance of optimizing surgeons’ sleep, nutrition, and physical activity. These foundational elements correspond to the physiological and biological needs described in Maslow's hierarchy of needs. According to Maslow, meeting these basic needs is essential for individuals to thrive at higher levels of well-being across multiple domains, including safety, social connection, esteem, and self-actualization (2).

## Surgical burnout: dimensions and implications

Burnout has become a major concern in surgery, affecting both trainees and practicing surgeons. Despite being a well-recognized concern for many years, it remains unclear whether burnout is truly increasing or simply plateauing.

In 2014, Elmore et al. conducted a national survey on burnout among U.S. general surgery residents, using the Maslach Burnout Inventory to assess emotional exhaustion, depersonalization, and personal accomplishment (3). They found that 69% of residents met burnout criteria in at least one domain, with higher rates observed among women, those planning to enter private practice, and those working longer hours.

Burnout is not limited to trainees but also affects practicing surgeons. A 2008 American College of Surgeons survey reported high burnout rates, linked to younger age, having children, specialty (trauma surgeons most affected), on-call frequency, work-life integration conflicts and inadequate compensation (4, 5). Additional stressors included keeping up with regulations, electronic health records, technological advances, patient expectations, and legal concerns.

In a follow-up survey of 7,905 surgeons, 6% reported suicidal ideation in the previous year, though only 26% sought psychiatric help due to license concerns (5).

More recently, a 2018 survey of six general surgery programs in North Carolina revealed that 75% of residents and faculty experienced burnout, with 39% meeting depression criteria and 12% reporting recent suicidal thoughts. Barriers to appropriate selfcare included lack of time, denial, and stigma, leading many surgeons to advise against surgical careers for their children (5, 6). Meta-analytic evidence reinforces the high prevalence of burnout.

Gili-Miner et al. analyzed 21 cross-sectional studies involving 3,325 general surgeons and reported that 43% experienced emotional exhaustion (EE) and 41% depersonalization (DP). Although the analysis explored potential moderators, significant heterogeneity remained unexplained, suggesting that burnout arises from a complex interplay of personal, professional, and systemic factors. No publication bias was detected, supporting the robustness of the findings (7).

Trauma surgeons appear to be particularly vulnerable. A systematic review and meta-analysis of 19 studies involving 4,634 trauma surgeons reported a pooled burnout prevalence of 60%, markedly higher than most other surgical specialties. Emotional exhaustion and depersonalization were especially prominent, yet personal accomplishment remained relatively preserved, suggesting inherent resilience factors in this specialty. Younger age, long working hours, and administrative burden emerged as major contributors to burnout, whereas mentorship and protected non-clinical time appeared protective (8).

A systematic review of 103 studies including 63,587 surgeons and trainees in the US and Canada found that 41% of surgeons overall met criteria for burnout, with trainees more affected than attending surgeons (46% vs. 36%). According to Etheridge JC et al, over the 25-year period analyzed (1996–2021), burnout prevalence remained largely stable, showing a slight, non-significant decrease (−4.8% per decade). Emotional exhaustion and depersonalization scores declined modestly over time, while personal accomplishment scores remained unchanged. Despite adjustments for specialty, training status, practice setting, and study quality, high heterogeneity persisted across the studies. These findings suggest that although burnout may not be increasing, it remains at unacceptably high levels, emphasizing the ongoing need for targeted interventions across all surgical training levels and specialties (9).

Conversely, a less optimistic review from Golisch KB et al. highlights that burnout in surgery remains a significant and growing concern, disproportionately affecting younger and female surgeons. Contributing factors include poor work-life balance, long working hours, and workplace mistreatment. Despite structured interventions to reduce dissatisfaction and emotional exhaustion, rates continue to rise. According to the authors several surveys indicate that 35%–50% of attending surgeons and up to 70% of surgical trainees experience burnout, with stress and distress levels 2–5 times higher than in the general population. Trauma surgeons are particularly affected, with only 43% satisfied with work-life balance and 61% reporting burnout symptoms. Collectively, these data highlight that surgical burnout is a widespread, systemic issue requiring targeted interventions (10). The well-being of surgeons may also influence trainees. A recent French survey examined the quality of life (QoL) of senior surgeons and medical students in visceral surgery. Among 106 respondents, students reported slightly lower traumatic stress scores than senior surgeons, yet their overall mental health and burnout levels were positively affected by their supervisors’ well-being. Notably, the highest mental health scores among senior surgeons were associated with improved mental health and reduced burnout in students, highlighting the importance of role modeling and supervisory support (11).

Over the past 20 years, fewer U.S. medical students have chosen surgery, with international graduates filling many residency spots. The causes are multifactorial.

Generational differences play a role: while Baby Boomers (1946–1959) prioritized career success, members of Generation X (1960–1980) and Y (1981–2000) seek work-life balance, which is challenging in surgery due to long hours, physical demands, extended training, limited autonomy, and productivity pressures. Sanfey et al. demonstrated that lifestyle and family now heavily influence career choice, particularly among women, partially explaining declining interest in surgery (12, 13). As reported in several reviews, gender-related factors significantly influence the evolution of surgical careers. Although women now represent a growing proportion of medical students and surgical trainees in many countries, female surgeons continue to encounter structural barriers, including disparities in mentorship, leadership representation, and operative autonomy. In addition, challenges related to work–family integration remain particularly relevant, especially regarding the freedom to pursue motherhood, dedicate time to childcare, and maintain family and affective relationships. These factors may further contribute to discouraging some young physicians from pursuing long-term careers in surgery (14).

## Addressing burnout in surgery

Burnout among surgeons is a critical public health issue, affecting trainees and attending physicians alike. Emotional exhaustion, depersonalization, and reduced personal accomplishment result from demanding mental, emotional, and physical workloads, exacerbated by long hours, administrative burdens, workplace mistreatment, and poor work-life integration. These pressures negatively impact individual well-being, patient safety, team dynamics, and workforce sustainability (15). Addressing burnout cannot fall solely on individuals. Responsibility lies with healthcare systems, institutions, and professional organizations, as highlighted by Shanafelt et al. (15, 16).

More recently, the National Academy of Medicine's National Plan for Health Workforce Well-Being outlined a national framework for integrated action, engaging hospitals, academic centers, professional societies, insurers, and government entities to create a collaborative ecosystem for clinician well-being (17).

Sustainable improvements require coordinated action across all levels. Individual-level interventions, resilience training, mindfulness, emotional intelligence development, and self-compassion programs, have been shown to reduce psychological distress and improve professional motivation, personal accomplishment, and overall well-being (18–20). However, systemic support is equally crucial. Hospitals and training programs that implement wellness initiatives, mentorship programs, protected time policies, and administrative support can reduce burnout and attrition (18, 21). Leadership endorsement ensures engagement and reinforces the value placed on surgeon well-being. Emerging evidence emphasizes the impact of surgeons’ personal relationships on burnout. Work-home conflicts, spousal stress, and higher divorce rates are associated with burnout, highlighting the need for interventions that support relationships. These include surgeon-controlled strategies (e.g., dedicated family time) and organization-controlled measures (e.g., flexible childcare, parental leave, part-time work, mid-career retraining, couple workshops) (22–24). Delegating administrative tasks further preserves professional efficacy. National-level leadership that models well-being, promotes mentorship, enforces anti-bullying policies and advocates systemic change is essential for effective, sustainable implementation ensuring that interventions are implemented rigorously and consistently (25).

## Surgical enrollment in Italy

Recent trends in surgical training in Italy present a mixed picture. Until last year, fewer than 50% of residency positions in General Surgery were filled, and approximately 20% of enrolled residents discontinued their training.

According to data from the Italian Ministry of University and Research (MUR), across the national territory, 718 training positions in General Surgery were made available in 2024 and 662 in 2025 (D.M. n. 1589/2024 and D.M. n. 642/2025), but a substantial proportion of these positions remained unfilled, further underscoring the persistent difficulty in attracting sufficient numbers of trainees to the specialty.

These figures raise serious concerns in a system already strained by high retirement rates and longstanding shortages of surgical personnel across Italian hospitals. Without sufficient recruitment and retention, the national healthcare system (SSN) risks being unable to replace its surgical workforce or meet future clinical demands.

Beyond structural challenges, the social prestige of surgical career in Italy has declined. Once regarded as figures of technical mastery and moral authority, surgeons are now often seen as overworked, legally vulnerable and inadequately compensated, potentially discouraging younger generations from pursuing the specialty.

Residents in General Surgery training programs in Italy lament insufficient operative exposure, with limited opportunities to act as primary surgeon and substantial variability in surgical volume across training centers. Working conditions are often perceived as unsustainable, characterized by long hours, insufficient rest after night shifts, and a high risk of fatigue and burnout. Moreover, a substantial portion of their workload consists of non-educational task, such as administrative duties and frequent on-call shifts, which frequently overshadow structured learning. Residents also highlight limited medico-legal protection, noting unclear responsibilities during training and insufficient safeguards in the event of litigation. They further report that the economic compensation is inadequate compared to the workload and level of responsibility. Finally, career prospects are perceived as uncertain, with significant difficulties in securing stable positions and a frequent need for geographic mobility.

In the most recent selection cycle, however, a partial reversal of trend has emerged: preliminary enrolments in General Surgery have increased to about 63% (247/662 position unfilled, 2025 ANAAO ASSOMED – ALS).

Several factors may explain this improvement. Contributing factors likely include technological advances, particularly minimally invasive surgery and robotics, which make practice more modern, stimulating, and ergonomically favorable, as well as recent reforms in medical liability legislation, which have reduced perceived legal risk.

## Conclusion

The crisis of surgical vocation and burnout in surgery represent a pervasive and urgent challenge that threatens both surgeon well-being and the long term sustainability of the surgical workforce. Addressing this issue requires a structured, multilevel strategy integrating individual resilience-building, supportive institutional environments, and coordinated leadership at the national and professional-society level. Enhancing the attractiveness of surgical careers will depend on the ability of universities, training programs, and healthcare systems to develop educational and working environments that combine technical excellence with sustainable working conditions. In countries with publicly funded healthcare systems, physician remuneration should be reconsidered and potentially increased to better reflect workload, professional responsibility, and the long duration and intensity of surgical training.

Targeted interventions are particularly critical in light of the declining interest in general surgery and the growing shortage of trained surgeons worldwide. Ultimately, safeguarding the future of surgery will require reimagining surgical training and professional pathways to ensure that surgeons can achieve both clinical excellence and long-term professional well-being.

## References

[1] Patti MG Kibbe MR. Surgery 2019: an existential crisis. Updates Surg. (2019) 71(2):201–3. 10.1007/s13304-019-00664-x PMID: 31228103. 31228103

[2] Park J McElveen K. Optimal self-care for surgeons: sleep, diet, and exercise. Am Surg. (2025) 91(1):161–4. 10.1177/00031348241269422 PMID: 39120488. 39120488

[3] Elmore LC Jeffe DB Awad JL Turnbull MM R I. National survey of burnout among US general surgery residents. J Am Coll Surg. (2016) 223(3):440–51. 10.1016/j.jamcollsurg.2016.05.014 PMID: 27238875; PMCID: PMC5476455. 27238875 PMC5476455

[4] Dyrbye LN West CP Shanafelt TD. Defining burnout as a dichotomous variable. J Gen Intern Med. (2009) 24(3):440. author reply 441. 10.1007/s11606-008-0876-6 PMID: 19130150; PMCID: PMC2642563. 19130150 PMC2642563

[5] Shanafelt TD Balch CM Bechamps GJ Russell T Dyrbye L Satele D Burnout and career satisfaction among American surgeons. Ann Surg. (2009) 250(3):463–71. 10.1097/SLA.0b013e3181ac4dfd PMID: 19730177. 19730177

[6] Williford ML Scarlet S Meyers MO Luckett DJ Fine JP Goettler CE Multiple-Institution comparison of resident and faculty perceptions of burnout and depression during surgical training. JAMA Surg. (2018) 153(8):705–11. 10.1001/jamasurg.2018.0974 PMID: 29800976; PMCID: PMC6584717. 29800976 PMC6584717

[7] Gili-Miner M Giner L Gili-Ortiz E Franco-Fernández D Cabanillas-Moruno JL Germán-Castellani G Prevalence of burnout dimensions among general surgeons. A systematic review and meta-analysis. Cir Esp (Engl Ed). (2025) 103(10):800186. 10.1016/j.cireng.2025.800186 40701428

[8] Kirdar-Smith S Knight A Twumasi R. Burnout among trauma surgeons: a systematic review and meta-analysis. Trauma Surg Acute Care Open. (2025) 10(4):e001873. 10.1136/tsaco-2025-001873 PMID: 41170404; PMCID: PMC12570958. 41170404 PMC12570958

[9] Etheridge JC Evans D Zhao L Ibrahim N Wick EC Freischlag JA Trends in surgeon burnout in the US and Canada: systematic review and meta-regression analysis. J Am Coll Surg. (2023) 236(1):253–65. 10.1097/XCS.0000000000000402 PMID: 36519921. 36519921

[10] Golisch KB Sanders JM Rzhetsky A Tatebe LC. Addressing surgeon burnout through a multi-level approach: a national call to action. Curr Trauma Rep. (2023) 9(2):28–39. 10.1007/s40719-022-00249-x PMID: 36688090; PMCID: PMC9843106. 36688090 PMC9843106

[11] Venara A Annweiler C Gohier B Hamel JF. The quality of life of students is impacted by the quality of life of senior surgeons in French university hospitals. Langenbecks Arch Surg. (2025) 411(1):4. 10.1007/s00423-025-03873-8 PMID: 41191143; PMCID: PMC12589228. 41191143 PMC12589228

[12] Sanfey HA Saalwachter-Schulman AR Nyhof-Young JM Eidelson B Mann BD. Influences on medical student career choice: gender or generation? Arch Surg. (2006) 141(11):1086–94. discussion 1094. 10.1001/archsurg.141.11.1086 Erratum in: Arch Surg. (2007) 142(2):197. PMID: 17116801. 17116801

[13] Shanafelt TD West CP Sinsky C Trockel M Tutty M Wang H Changes in burnout and satisfaction with work-life integration in physicians and the general US working population between 2011 and 2023. Mayo Clin Proc. (2025) 100(7):1142–58. 10.1016/j.mayocp.2024.11.031 PMID: 40202475. 40202475

[14] Nottberg VI Klingelhöfer L Schweitzer NI Keller-Yamamura S Hackert T Molwitz I Structural barriers to work-family reconciliation in surgery: a gendered analysis of career disruption and care responsibilities. Updates Surg. (2026). 10.1007/s13304-025-02489-3 41649725 PMC13212764

[15] West CP Dyrbye LN Shanafelt TD. Physician burnout: contributors, consequences and solutions. J Intern Med. (2018) 283(6):516–29. 10.1111/joim.12752 PMID: 29505159. 29505159

[16] West CP Dyrbye LN Erwin PJ Shanafelt TD. Interventions to prevent and reduce physician burnout: a systematic review and meta-analysis. Lancet. (2016) 388(10057):2272–81. 10.1016/S0140-6736(16)31279-X PMID: 27692469. 27692469

[17] National Plan for Health Workforce Well-being. The National Academies Press (2022). 10.17226/26744

[18] Kratzke IM Barnhill JL Putnam KT Rao S Meyers MO Meltzer-Brody S Self-compassion training to improve well-being for surgical residents. Explore (NY). (2023) 19(1):78–83. 10.1016/j.explore.2022.04.008 PMID: 35534424. 35534424

[19] Lebares CC Coaston TN Delucchi KL Guvva EV Shen WT Staffaroni AM Enhanced stress resilience training in surgeons: iterative adaptation and biopsychosocial effects in 2 small randomized trials. Ann Surg. (2021) 273(3):424–32. 10.1097/SLA.0000000000004145 PMID: 32773637; PMCID: PMC7863698. 32773637 PMC7863698

[20] Lebares CC Hershberger AO Guvva EV Desai A Mitchell J Shen W Feasibility of formal mindfulness-based stress-resilience training among surgery interns: a randomized clinical trial. JAMA Surg. (2018) 153(10):e182734. 10.1001/jamasurg.2018.2734 PMID: 30167655; PMCID: PMC6233792. 30167655 PMC6233792

[21] Shen MR Zhuo L Madison K Bredbeck BC Kemp MT Santos-Parker JR How we do it: an innovative general surgery mentoring program. J Surg Educ. (2022) 79(5):1088–92. 10.1016/j.jsurg.2022.04.004 PMID: 35581113. 35581113

[22] Castillo-Angeles M Smink DS Rangel EL. Perspectives of general surgery program directors on paternity leave during surgical training. JAMA Surg. (2022) 157(2):105–11. 10.1001/jamasurg.2021.6223 PMID: 34851404; PMCID: PMC8637392. 34851404 PMC8637392

[23] Livingston-Rosanoff D Shubeck SP Kanters AE Dossett LA Minter RM Wilke LG. Got milk? Design and implementation of a lactation support program for surgeons. Ann Surg. (2019) 270(1):31–2. 10.1097/SLA.0000000000003269 PMID: 30921050; PMCID: PMC7474976. 30921050 PMC7474976

[24] Glavin RE Kent EM Frick SL Silver JK. Optimal well-being interventions for surgeons: beyond physiologic needs evidence-based recommendations and emerging novel solutions to reduce burnout. Am Surg. (2025) 91(12):2173–8. 10.1177/00031348251358441 PMID: 40642819. 40642819

[25] Bilimoria KY. Association for academic surgery presidential address-fanning the burnout fire: how our misconceptions and good intentions could fail Tomorrow's Surgeons. J Surg Res. (2021) 257:A1–A11. 10.1016/j.jss.2020.06.005 PMID: 32768197. 32768197

